# A Microfluidic Chip for Studies of the Dynamics of Antibiotic Resistance Selection in Bacterial Biofilms

**DOI:** 10.3389/fcimb.2022.896149

**Published:** 2022-05-10

**Authors:** Po-Cheng Tang, Olle Eriksson, Josefin Sjögren, Nikos Fatsis-Kavalopoulos, Johan Kreuger, Dan I. Andersson

**Affiliations:** ^1^ Department of Medical Biochemistry and Microbiology, Uppsala University, Uppsala, Sweden; ^2^ U-Print, Uppsala University 3D-Printing Facility, Uppsala University, Uppsala, Sweden; ^3^ Corline Biomedical Aktiebolag (AB), Uppsala, Sweden; ^4^ Department of Medical Cell Biology, Uppsala University, Uppsala, Sweden

**Keywords:** biofilm, microfluidics, antibiotic resistance, evolution, *Escherichia coli*.

## Abstract

Biofilms are arguably the most important mode of growth of bacteria, but how antibiotic resistance emerges and is selected in biofilms remains poorly understood. Several models to study evolution of antibiotic resistance have been developed, however, their usability varies depending on the nature of the biological question. Here, we developed and validated a microfluidic chip (Brimor) for studying the dynamics of enrichment of antibiotic-resistant bacteria in biofilms using real-time monitoring with confocal microscopy. *In situ* extracellular cellulose staining and physical disruption of the biomass confirmed *Escherichia coli* growth as biofilms in the chip. We showed that seven generations of growth occur in 16 h when biofilms were established in the growth chambers of Brimor, and that bacterial death and growth rates could be estimated under these conditions using a plasmid with a conditional replication origin. Additionally, competition experiments between antibiotic-susceptible and -resistant bacteria at sub-inhibitory concentrations demonstrated that the antibiotic ciprofloxacin selected for antibiotic resistance in bacterial biofilms at concentrations 17-fold below the minimal inhibitory concentration of susceptible planktonic bacteria. Overall, the microfluidic chip is easy to use and a relevant model for studying the dynamics of selection of antibiotic resistance in bacterial biofilms and we anticipate that the Brimor chip will facilitate basic research in this area.

## 1 Introduction

Biofilms are communities of bacteria that are attached either to a surface or to each other and embedded within a self-produced matrix of extracellular polymeric substances (EPS) (Flemming et al., 2016; Flemming and Wuertz, 2019). Biofilms are less susceptible to antibiotics and biocides representing a serious challenge for effective treatment of a wide range of infections, and in particular for biofilm-colonized medical devices.

Evolution of resistance to antibiotics is dependent on the interplay between genetic and phenotypic resistance mechanisms. Evolution at high antibiotic levels (above the minimal inhibitory concentration, MIC) has been extensively studied and it is only recently that low, non-lethal levels of antibiotics (sub-MIC levels) have been thoroughly examined (Gullberg et al., 2011; Liu et al., 2011a; Gullberg et al., 2014; Lundström et al., 2016; Khan et al., 2017; Wistrand-Yuen et al., 2018). Importantly, antibiotic levels far below the MIC can impose a selective pressure for the development of resistance, thereby contributing to the evolution of antibiotic resistance (Oz et al., 2014). Of relevance in this context is the minimal selective concentration (MSC), which is defined as the lowest concentration of antibiotic at which a resistant bacterium will outcompete its susceptible counterpart and become enriched within a population (Gullberg et al., 2011; Andersson and Hughes, 2014). Apart from *bona fide* antibiotic resistance mechanisms, bacterial biofilms can also show antibiotic tolerance by phenotypic mechanisms such as reduced ability of antibiotics to penetrate biofilms and the presence of dormant cells in the inner regions of the biofilms as well as cooperative behaviors between members of the biofilm population, adding another layer of complexity (Flemming and Wingender, 2010; Nadell et al., 2016; Ciofu et al., 2017; Koo et al., 2017; Arciola et al., 2018; Crabbé et al., 2019).

The properties of bacterial biofilms can generally not be extrapolated from our understanding of planktonic lifestyle (Flemming et al., 2016). Thus, several methods for biofilm studies have been developed, such as the MBEC™ assay (based on the Calgary biofilm device), the rotating disk reactor, the CDC biofilm reactor, and the colony biofilm model and more advanced technologies utilizing high-resolution microscopy, hydrogels and microfluidics. These methods can largely be grouped into three: open-, closed-, or mixed-systems. Closed-systems are commonly used owing their simplicity and ease of use. Single- or mixed-species bacteria are incubated together in microtiter well plates and biofilm formation occur for a certain time under static conditions (Zaborskytė et al., 2022). The system does not have constant supply of fresh nutrients essential for bacterial growth, leading to consumption of nutrients and accumulation of bacterial metabolic waste products. Furthermore, microenvironmental changes in the culture conditions may result in stochastic variations in these systems prompting the recent requirement for a minimum information guideline when using closed-systems (Kragh et al., 2019; Allkja et al., 2020). Open-systems address this limitation with continuous supply of fresh nutrients to support continuous growth of biofilms. Typically a flow of culture medium, with or without bacteria, is generated using a peristaltic or syringe pump to for example simulate the conditions during urinary tract infections (Hong et al., 2012; Hol and Dekker, 2014; Azeredo et al., 2017; Yan et al., 2017; Pousti et al., 2019; Heyman et al., 2020). Mixed-systems combine the ease-of-use of close-systems and address the limitation of nutrient supply by a nutrient replenishment and waste discarding step at specific intervals during biofilm cultivation and propagation (Poltak and Cooper, 2011; Zaborskytė et al., 2022).

Microfluidic approaches coupled with advanced live imaging provides a platform where it is possible to study biofilms *in situ* under different hydrodynamic conditions (Kheyraddini Mousavi et al., 2012; Yawata et al., 2016; Azeredo et al., 2017) and at high-resolution (Baltekin et al., 2017; Wistrand-Yuen et al., 2020). Previously developed microfluidic chips for biofilm studies feature straight flow channels (Benoit et al., 2010; Rusconi et al., 2014; Liu et al., 2015; Siryaporn et al., 2015; Massalha et al., 2017; Zarabadi et al., 2017; Chu et al., 2018; Martinez-Corral et al., 2018; Wucher et al., 2019) or complex geometries to generate intricate flow paths (Liu et al., 2011b; Rusconi et al., 2011; Coyte et al., 2017; Dehkharghani et al., 2019). These flow systems suffer from randomness with respect to the location for biofilm formation in the devices, and difficulty in harvesting biofilms with precision. Specifically, there are several microfluidic chips developed for antibiotic resistance studies (Kim et al., 2010; Straub et al., 2020; Zoheir et al., 2021). However, the current devices are either sensitive towards formation of air bubbles which disrupt the cultivation of biofilms on chip or could be sensitive towards changes in the antibiotic concentration gradient generated as a result of biofilm cultivation. The former is a common burden in most microfluidic applications, as they can severely alter the flow characteristics of fresh nutrient and removal of wastes from the biofilm. The likelihood of this phenomenon increases with prolonged incubation and operation time. Therefore, you can expect a microenvironmental change due to the alternating presence and absence of the air bubbles. The latter has not been explored with the devices proposed. In principle, these chips could be applied for studies of antibiotic resistance selection, however, this is on the basis that (i) the images could be captured over time without influencing the concentration gradient generated in the chip, (ii) the seeded cells are allowed to grow over the entire surface of the chip without any issues of clogging and (iii) biofilm cultivation should not influence the sensitive gradient-generation flow system. Recently, laser capture microdissection was used to extract a small subset of cells at different regions within a cryo-embedded biofilm (Pérez-Osorio and Franklin, 2008), but this particular method kills the cells preventing the generation of new daughter biofilms, which is often needed to address evolutionary questions (Martin et al., 2016; Santos-Lopez et al., 2019; Thibault Stalder et al., 2020).

Here, we describe the development of a new *in vitro* microfluidic chip called Brimor (the letter B stands for biofilms and *rimor* is the Latin word meaning to probe, search or explore) which allow for specific and reproducible isolation of distinct biofilm sections whilst maintaining the spatial structure of the biofilm. Single-use, disposable Brimor microfluidic chips were easily fabricated at low-cost using 3D-printed molds (encoding fluidic channels), polydimethylsiloxane (PDMS) casting, and bonding of the resultant PDMS replica piece to a glass slide. Together with the basic components of a microfluidic system, the entire system enabled controlled cultivation of bacterial biofilms. We utilized *Escherichia coli* for biofilm formation with *in situ* staining to confirm the presence of extracellular cellulose in the biofilms. By live imaging and alterations in flow-rate, we showed that seeded planktonic cells shift towards a biofilm state in the microfluidic chip. We further demonstrated the novel capability of the system for controlled harvesting of defined layers of the cultivated biofilms. Finally, the new biofilm model was used to measure growth and death rates of *E. coli* during biofilm formation and to determine the minimal selection concentration in biofilms (MSCB) when *E. coli* biofilms were exposed to ciprofloxacin. The results showed that exposure to very low non-inhibitory ciprofloxacin concentrations can enrich for resistant mutants in bacterial biofilms.

## 2 Materials and Methods

### 2.1 Design and Fabrication of the Microfluidic Chip Brimor

The different microfluidic chip designs were drawn using the Autodesk Fusion 360 (Autodesk) software v.2.0.8176. All fluidic channels were 100 µm high, ranged from 200 to 400 µm in width and ranged from 3 to 4 mm in length (see **Supplementary Material** for stereolithography (.stl) file). Molds were printed using a Form 2 3D-printer (Formlabs, Somerville, MA, USA) using black resin (v.3 & v.4, Formlabs) with 25 µm thick layers as previously described (Hernández Vera et al., 2019; Wistrand-Yuen et al., 2020). Microfluidic chips were generated by PDMS (at 10:1 ratio of liquid elastomer to curing agent; Sylgard 184, DowSil, Sweden) casting into the various 3D-printed molds (Duffy et al., 1998) to generate negative PDMS replica. Briefly, air bubbles were removed from the PDMS by degassing for 25 min at room temperature before curing of the PDMS at 80°C for 45 min. Thereafter, the PDMS was allowed to cool in the mold to room temperature overnight. The resultant polymerized PDMS with microfluidic structures was peeled off from the mold and bonded to a microscope glass slides (75 x 25 x 1 mm, SuperFrost Plus, VWR, Sweden) after surface activation for 1 min using an oxygen corona plasma treater (BD20-AC, ETP, Chicago, IL, USA) as previously described (Haubert et al., 2006; Wistrand-Yuen et al., 2020) before being sandwiched between two aluminum plates held together by a clamp and baked for 45 min at 80°C. Resultant chips were allowed to cool in a sterile petri dish to room temperature 16-18 h prior to use (**Supplementary Figure S1** and **Supplementary Movie S1)**.

### 2.2 Bacterial Strains, Genetic Engineering, and Growth Conditions

All bacterial strains used in this study were derived from *Escherichia coli* K-12 strain MG1655 and are listed in **Supplementary Tables S1**. Where required, a combination of generalized transductions with P1*vir* phage, λ-red, and duplication-insertion recombineering were performed as described previously for genetic engineering of the strains (Ikeda and Tomizawa, 1965; Näsvall et al., 2017). Briefly, this method consists of the generation of a tandem duplication using the *Acatsac1* cassette in the proximity of the mutated gene. An *Acatsac1* cassette was amplified with Phusion High-Fidelity DNA Polymerase (Thermo Fisher Scientific Inc.) with 40 nucleotide overhangs with homologies to the target region for duplication. The *Acatsac1* cassette was inserted in the mutant strains *via* λ-red recombination and the antibiotic resistance cassette was selected by plating cells on Luria-Bertani (LB) (Miller, Sigma-Aldrich, USA) agar plates supplemented with 12.5 mg/L chloramphenicol to generate a temporary duplication strain. The duplication was transduced back into the parental strain with the P1*vir* phage and plated on no salt LB agar plates containing 50 mg/L sucrose for segregation. The temporary duplications are unstable genetic modifications that resolve themselves by homologous recombination and leave the mutations of interest in the recipient strain without any scar sequence. PCR and Sanger sequencing (Eurofins Genomics) were performed to confirm that the intended mutations were correctly reconstructed. If not indicated otherwise, liquid and solid media used for growth were always LB broth and LB agar (LB broth supplemented with 1.5% (w/v) agar), respectively. Unless otherwise stated all strains were first grown overnight on LB agar at 37°C as single colonies from –80°C stock cultures, and then inoculated as a 10 mL preculture incubated with vigorous agitation (198 r.p.m) on an orbital shaker at 30 ± 2°C.

### 2.3 Simulations of Fluid Dynamics

Fluid dynamic simulations were carried out using the finite element COMSOL Multiphysics modelling software v.5.5 (Comsol, LA, CA, USA). 2D real-size microfluidic channel geometries were imported as.dxf files produced by Autodesk Fusion 360 software and utilized for extremely fine mesh construction. The geometry used for simulations included the growth chamber, and the inlet and outlet channels. Simulations were performed using the steady state Navier-Stokes model in stationary phase and the fluid was considered to have the same physical properties as water. No slip boundary conditions were set on the walls of the channels and a laminar flow with a mean velocity of 2.1e^-3^ m/s was set at the inlet and zero pressure was set at the outlet. Flow inside the chip was then calculated using the UMPACK solver.

### 2.4 Chip Operation

Liquid precultures (as described in previous section) were diluted to an optical density (OD_600_) = 0.15 or 0.075 (for single strain or mixed-strain experiments, respectively) in fresh LB broth. 11 µL of this dilution was immediately loaded into the inlet channel from the inlet port of the microfluidic chip until the outlet channel and the outlet port was filled with liquid. Thereafter, the chip was seated on a custom-made 3D-printed chip holder and inserted horizontally into a 50 mL centrifuge tube. Centrifugation was carried out at 800 × *g* for 6 min, with centrifugal acceleration and deceleration set to 6 (Megafuge 8, VWR, Sweden) to ensure cells are specifically placed at the bottom of the growth chamber area with no damages made to the chip. Thereafter, liquid to liquid connections were made between tubing connected to syringes filled with growth medium and the inlet ports of the biofilm chip. Briefly, prior to seeding of bacteria in the microfluidic chip, two 60 mL syringes (Luer-Lok, BD, USA) were filled with 30 mL of LB broth and connected *via* 24-gauge needles (Microlance 3, BD, USA) to a 90 cm tubing (Tygon ND-100-80, 0.020” × 0.060” OD, Cole-Parmer, USA). The two syringes were then placed in a syringe pump (Model 22, Harvard Apparatus, Holliston, MA, USA) with the flow rate set to 100 μL/min and allowed to run for 1 h prior to start of an experiment to ensure that no air bubbles were trapped in the tubing. To the respective outlet ports, a 60 cm tubing was connected leading to waste collection beakers. Once all tubing was connected to the chip, the flow rate was set to 100 µL/min for one min to ensure removal of non-specifically seeded cells remaining in the inlet and outlet channels. Immediately thereafter, the flow rate was set to 5 μL/min and the chip securely placed in a microscope stage slide holder. Finally, an external vacuum source was connected to the vacuum grid. All experiments described in this work were carried out at room temperature (25 ± 2°C).

### 2.5 Time-Lapse Microscopy

All time-lapse images for experiments which evaluated the effects of fluid flow in relation to biofilm growth in various chip designs and determination of the number of generations of E. coli biofilms were taken using an Axiovert 200 M fluorescence microscope with AxioVision software v.4.8.2 (Carl Zeiss, Germany). Additionally, harvesting of distinct partitions from the biofilms were also visualized in real-time and captured on the Axiovert microscope in combination with Debut Video Capture software v.2.16. All time-lapse imaging for *in situ* staining, partitioning, integrity, harvest and direct competition experiments were carried out using an inverted confocal microscope (Zeiss LSM 700, Carl Zeiss, Germany) with the Zen 2009 software v.14 (Carl Zeiss, Germany). All resultant time-lapse images for experiments were analyzed using Fiji (Schindelin et al., 2012) v.2.0.0-rc-65/1.52b.

### 2.6 Biofilm Partitioning and Harvest

Biofilms were cultivated for 16 ± 1 h or for 144 h (corresponding to 6 days) prior to biofilm partitioning. Briefly, the 16 h old biofilm was partitioned arbitrary into three sections. The flow rate was changed from 5 µL/min to 1000 µL/min for 10 sec before returning to the initial flow rate in order to harvest and collect the outermost biofilm layer. The second biofilm layer was partitioned by applying a flow rate of 1000 µL/min for 20 sec before returning to the initial flow rate to harvest and collect the layer. Thereafter, a flow rate >1999 µL/min for 10 sec was used to eject the remaining biofilm in the growth chamber. Similarly, the 144 h biofilm was partitioned and harvested into three arbitrary layers in a similar fashion, using a flow rate of 1000 µL/min for 47 sec to harvest the outermost biofilm layer. The remaining biofilm was harvested with a flow rate of >1999 µL/min using visual monitoring and separate collection of the two innermost biofilm layers in two equal halves.

### 2.7 *In Situ* Staining

For *in situ* staining experiments, strains DA5438, DA64255 and DA46932 was precultured (as described in the previous section) prior to seeding and cultivation in the chip for 16 ± 1 h at room temperature with fluorescence and differential interference contrast images (DIC; captured using the PMT detector) collected at 20 min intervals using a 5×/0.16 Plan-Apochromat objective lens. Images were saved with a frame scan mode of 1024 × 1024 pixels resolution as 16-bit with a line averaging of 4. For staining of resultant biofilms grown for 16 ± 1 h with calcofluor white (CFW), tubing connected to the LB growth medium syringe was removed by the needle head and connected to the needle head of syringes containing PBS supplemented with 34.3 g/L CFW M2R (Sigma-Aldrich, USA) syringe. Staining with CFW was carried out at a flow rate of 5 µL/min for 4 h and was collected at 10 min intervals. Thereafter de-staining with PBS was carried out for 4 h at a flow rate of 5 µL/min and images collected every 5 min. The microscope settings were kept constant for all imaging stages and false coloring of resultant fluorescent images was changed to from dark blue to negative images for better visualization. Time-lapse videos were generated at a frame rate of 7 frames per second with jpeg compression.

### 2.8 Biofilm Integrity by Flow Rate Changes

For biofilm integrity experiments, DA5438 and DA72167 strains was precultured (as described in the previous section) prior to seeding and cultivation in the chip for 16 ± 1 h at room temperature. Biofilm integrity between wild-type *E. coli* (DA5438) and quadruple deficient (DA72167) strains was examined by partitioning the biomass. This was achieved by increasing the flow rate from 5 µL/min to 100 µL/min until the entire biofilm was removed using visual monitoring. Images were captured for the duration of this process and all experiments were repeated independently three times.

### 2.9 Number of Generations and Death Event Estimations

To measure division rates and simultaneously estimate cellular death events from cultivated biofilms in the chip, we utilized the pAM34 plasmid in an *E. coli* strain (Frenoy and Bonhoeffer, 2018). pAM34 is a pBR322 ColE1 plasmid derivative with replication control under the IPTG inducible promoter pLac. ColE1 plasmids are nonconjugative and cannot be horizontally gene transferred between cells unless in the presence of another mobilizer plasmid. In the presence of IPTG, the plasmid is stably maintained and passed on from parental to daughter cells within a given population of bacterial cells. Conversely, the plasmid does not replicate in the absence of IPTG in the growth medium and is stochastically segregated when cells divide. We utilized the decrease in plasmid frequency between the start and end of biofilm growth within the chip to compute the number of bacterial cell divisions that occurred between these two time points and to estimate the relative death rate. Briefly, four independent experiments were started with the *E. coli* pAM34 carrying strain. The plasmid carrying strain was precultured (as described in previous section) in LB supplemented with 0.1 mM IPTG and 100 mg/L of ampicillin (to ensure the maintenance of pAM34 in the entire preculture population, see results section and Fig. 4 for more details). The culture was then diluted in fresh LB to an OD_600_ of either 0.15, 0.1, or 0.06 prior to seeding of four independent chips. Simultaneously, a sample of the diluted culture at different OD_600_ was plated at appropriate dilutions on two different LB agar mediums: LB, to count the total number of bacteria (with and without the plasmid), and LB agar supplemented with 0.1 mM IPTG and 100 mg/L ampicillin, to count the number of bacteria carrying a copy of the plasmid. Liquid volume dislodged from the centrifugation step as well as the chip flushing step during set-up of system was noted for calculations, collected and plated at appropriate dilutions on LB agar with and without IPTG and ampicillin as described above. The entire biofilm was harvested in growth medium by increasing the flow rate from 5 µL/min to >1000 µL/min with manual plunging of the syringe. The volume of the liquid that exited the connected outlet tubing were noted for calculations and samples collected. Samples were vortexed vigorously before plating at appropriate dilutions on the two different LB agar mediums (described above). All plates were incubated at 37°C overnight before counting colonies and determining the colony forming units (cfu). To estimate the cfu of cells remaining in a seeded chip, we subtracted the calculated cfu from the inoculum used for seeding of the chips to the calculated cfu dislodged from the centrifugation and flush steps during system set-up. This served as the initial estimated cfu prior to biofilm cultivation whilst the final cfu was calculated from the entire harvested biofilm. Three parameters were then computed derived from previously described mathematical models (Frenoy and Bonhoeffer, 2018). Briefly, the number of generations assuming no death (*g_no-death_*, Eq. 1) allowed us to estimate the rate of residual replication of the plasmid relative to the division rate in the absence of IPTG (*r*, Eq. 2). Residual replication of plasmid is defined as the small amount of plasmid that is still replicated in the absence of IPTG. This allowed us to compute the number of generations based on plasmid segregation (*g*, Eq. 3) and determine the relative death rate (temporal death rate divided by temporal division rate) (*d*, Eq. 4). Plasmid segregation is defined as plasmid replication through which identical copies of plasmids are produced and equally distributed among the newly produced daughter cells. These estimations were computed based on the cfu, where total number of cells (measured by plating on LB) at an initial timepoint (*N_i_*) and final time point (*N_f_*) and the number of cells bearing at least one copy of pAM34 at an initial timepoint (*F_i_*) and final time point (*F_f_*) (measured by plating on LB + ampicillin + IPTG).


(1) $$g_{no-death}=log_{2}\frac{N_{t}}{N_{0}}$$

(2) $$r=2^{\frac{log_{2}\frac{F_{f}}{F_{i}}}{log_{2}\frac{N_{f}}{N_{i}}}\;+\;1}-1$$

(3) $$g=\frac{log_{2}\frac{F_{t}}{F_{0}}}{log_{2}\frac{1+r}{2}}$$

(4) $$d=\frac{1-log_{2}\frac{N_{t}}{N_{0}}}{g}$$

### 2.10 Planktonic Minimal Inhibitory Concentration Determination

Minimal inhibitory concentrations (MICs) of the various bacterial strains cultivated planktonically were determined by broth microdilutions according to EUCAST, CLSI and ISO 20776-1:2006 guidelines. We reasoned liquid estimations of the MIC values were most representative of the microfluidic growth conditions. Briefly, bacterial cultures from –80°C were streaked on LB agar and incubated at 37°C for 16-18 h. Thereafter, colonies were suspended homogenously in 0.9% (w/v) NaCl to reach cell densities of 0.5 MacFarland. A stock solution of 10 g/L of ciprofloxacin (Sigma Aldrich) was prepared in 0.1 M HCl and diluted in LB broth to obtain a solution containing 8 mg/L of ciprofloxacin. This was then used to prepare serial dilutions of the antibiotic in a 96-well plate (Nunc, Thermo Scientific), each well contained 50 μL of the antibiotic solution. Fifty microliters of the 0.5 MacFarland bacterial suspension were inoculated in the wells containing the ciprofloxacin. Serial dilutions were prepared throughout the microtiter plate and the plate was incubated at 37°C for 16-20 h. The results were determined by visual inspection for bacterial growth and the MICs was read as the lowest concentration at which no cell growth was observed. A susceptible quality control (QC) *E. coli* ATCC 25922 strain was included in the assay.

### 2.11 Competitions in Bacterial Biofilms

For all direct competition experiments, each strain pair was separately precultured (as described in previous section) and then diluted in fresh LB to an OD_600_ = 0.15. The strain pairs were: DA52554 and CH367; DA52554 and CH368, DA66208 and CH367, with corresponding orange fluorescent protein (OFP) and cyan fluorescent protein (CFP), respectively. Each competing strain pair was mixed at a 1:1 ratio and diluted further to a final OD_600_ of 0.075 prior to seeding of the chip. Biofilms were cultivated in the chip for 16 ± 1 h at room temperature with fluorescence and differential interference contrast images (captured using the PMT detector) collected at 20 min intervals with a 5×/0.16 Plan-Apochromat objective lens. Fluorescent proteins were excited with 405 nm and 488 nm laser lines for CFP and OFP, respectively. Images were saved with a frame scan mode of 1024 × 1024 pixels resolution as 16-bit with a line averaging of 4. Resultant images were analyzed with the initial background subtracted using a rolling ball background subtraction with a 50-pixel radius. Thereafter, regions of interest were defined using the Fiji rectangular selection tool to limit the analysis only within the biofilm growth chamber. Normalized fluorescence was determined for 16 h (resulting in 49 time points) by dividing the resultant arbitrary fluorescence intensity units (AFIU) across the 49 time points with the initial AFIU for each independent experimental set-up. Thereafter, the ratio of resistant to susceptible population from the corresponding fluorescent protein in the strains was determined from these normalized AFIU and the selection coefficient (*s*) calculated using the regression model (Eq. 5) as previously described (Dykhuizen, 1990), where *R* represents the ratio of resistant to susceptible across the different time points.


(5) $$s=\frac{ln\frac{R_{t}}{R_{0}}}{t}$$

To determine the effects of low concentrations of ciprofloxacin on cultivated biofilms, a minimum of three independent competitions were performed at the planktonic MIC and four at sub-MIC levels. For each independent competition, the left growth chamber of the chip was exposed to LB supplemented with varying concentrations of ciprofloxacin, whilst the right growth chamber of the chip was exposed to LB only. In most experiments, the resulting biofilm biomass was uniform. There were some experiments which had higher biomass, likely a result of the fluctuation in the incubation temperature or due to seeding and handling steps in the set-up and operation protocol. We reasoned that these differences did not affect the calculated resistant and susceptible population ratios. To validate the observed ratios from microscopy, the entire biofilm from 4 independent chips were harvested and collected at 0, 4, 8, and 16 h (as described in number of generations and death event estimations section) and plated on LB agar and LB supplemented with 0.15 mg/L ciprofloxacin agar to determine the resistant and susceptible ratios *via* traditional agar plating and counting the number of colonies observed to determine the cfu. To account for the genetic differences between the parental strains and inert differences in the OFP and CFP markers used, a competition between the two strains tagged with either CFP or OFP (CH367 and DA52554, respectively) were performed in three independent experiments. The mean *s* values from these were used to compensate for the calculated s values from each independent competition experiments with and without ciprofloxacin exposure.

## 3 Results

### 3.1 Design of the Biofilm Chip and System Overview

**Figure 1:**
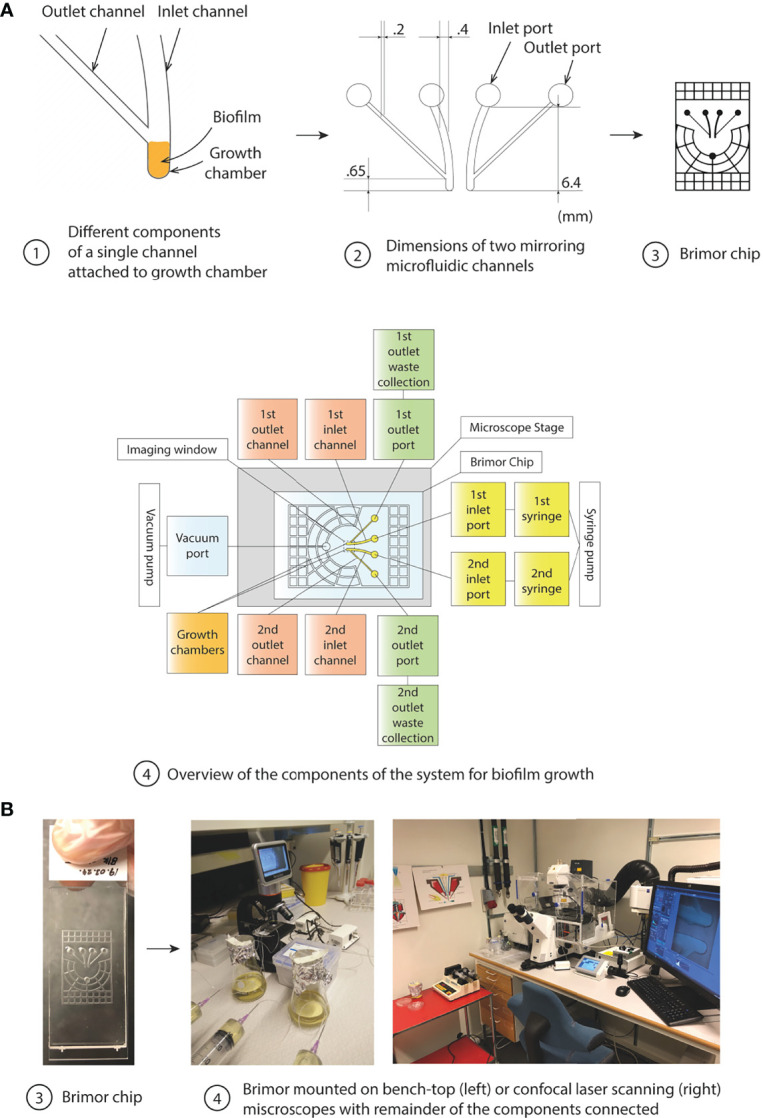
Overview of the components of the microfluidic system for biofilm studies. **(A)** Illustrations indicating key features of the microfluidic chip. (1) The inlet flow channel, the biofilm growth chamber, and the outlet flow channel positioned with a 45° angle in relation to the inlet channel of the microfluidic chip is shown. Cells (represented by orange) proliferate and form a biofilm in the growth chamber which eventually fill up the entire volume of the growth chamber before cells (or cell aggregates) are efficiently removed from the outer part of the biofilm due to the high fluid sheer stress in the flow channels, (2) Dimensions and relative positioning of the flow channels and the two growth chambers in a chip, (3) Overview of the complete Brimor chip showing the vacuum grid that surrounds the centrally positioned flow channels and growth chambers, (4) Schematic overview of the complete system. **(B)** Photographs of the (3) Brimor chip and (4) complete system operated with a simple light bench-top microscope (left) or with a confocal microscope (right).

**Figure 2:**
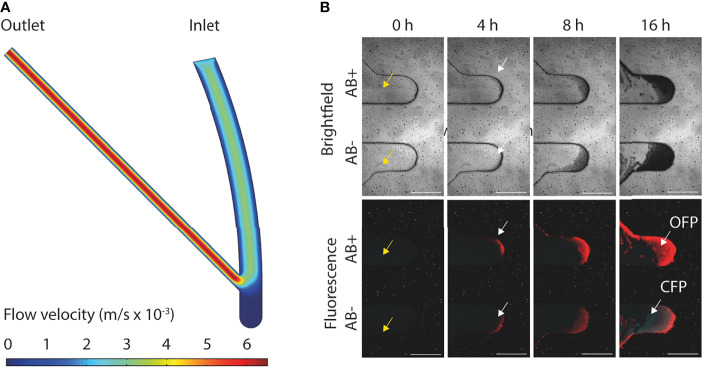
Overview of flow dynamics and formation of *E. coli* MG1655 biofilms. **(A)** FEM simulation of the flow velocities (m/s × 10^-3^) in the flow channels and growth chamber of the biofilm chip. **(B)** Representative confocal images of mixed strain biofilm development with (AB+) or without (AB-) ciprofloxacin treatment at inhibitory concertation (0.16 mg/L). CFP (cyan fluorescent protein) and OFP (orange fluorescent protein) tagged *E. coli* MG1655 both susceptible to ciprofloxacin were cultivated in the bottom growth chamber in the microfluidic chip. OFP tagged *E. coli* MG1655 resistant to ciprofloxacin and CFP tagged *E. coli* MG1655 susceptible to ciprofloxacin were cultivated in the top growth chamber in the microfluidic chip. Yellow arrows indicate absence of cells within the inlet and outlet channels after biofilm seeding. White arrows indicate initial biofilm formation at 4 h post-seeding. All scale bars represent 500 µm.

**Figure 3:**
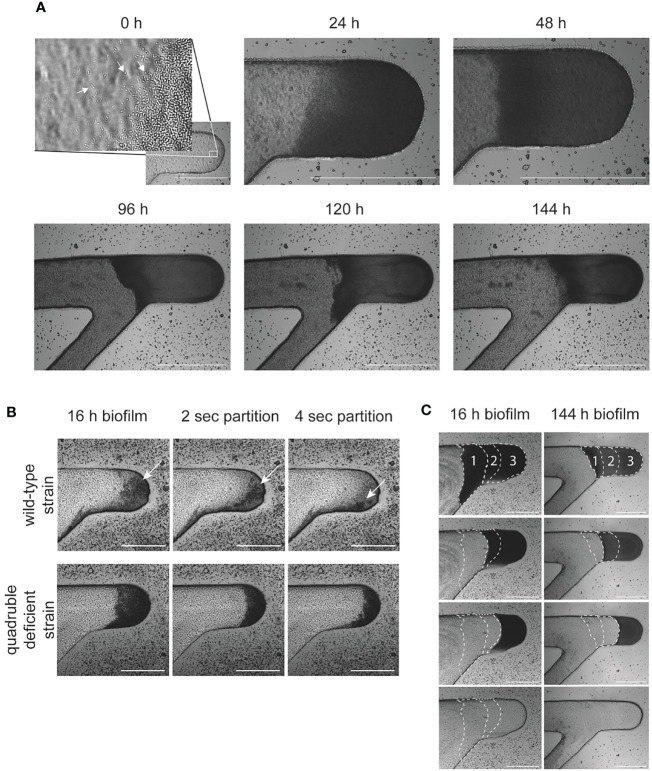
Formation of *E. coli* MG1655 biofilms. **(A)** Formation of biofilm over 6 days in the microfluidic chip. Directly after seeding (0 h) individual pelleted cells were observed at the bottom of the growth chamber. White arrows indicate single and actively dividing seeded cells. **(B)** The integrity of 16 h cultures were evaluated to determine biofilm formation. Biofilm formation by wild-type *E. coli* (MG1655) was compared to an isogenic quadruple deficient strain lacking the entire genetic pathway for cellulose, curli, colonic acid and fimbriae synthesis. **(C)** The microfluidic chip enabled stratification and harvesting of biofilms, examples of controlled removal of biofilms grown for 16 h or 6 days are shown. Biofilms were divided into three sections (indicated by white dotted lines) and stratified in sequential sequence (see *Materials and Methods*). All scale bars represent 500 µm.

**Figure 4:**
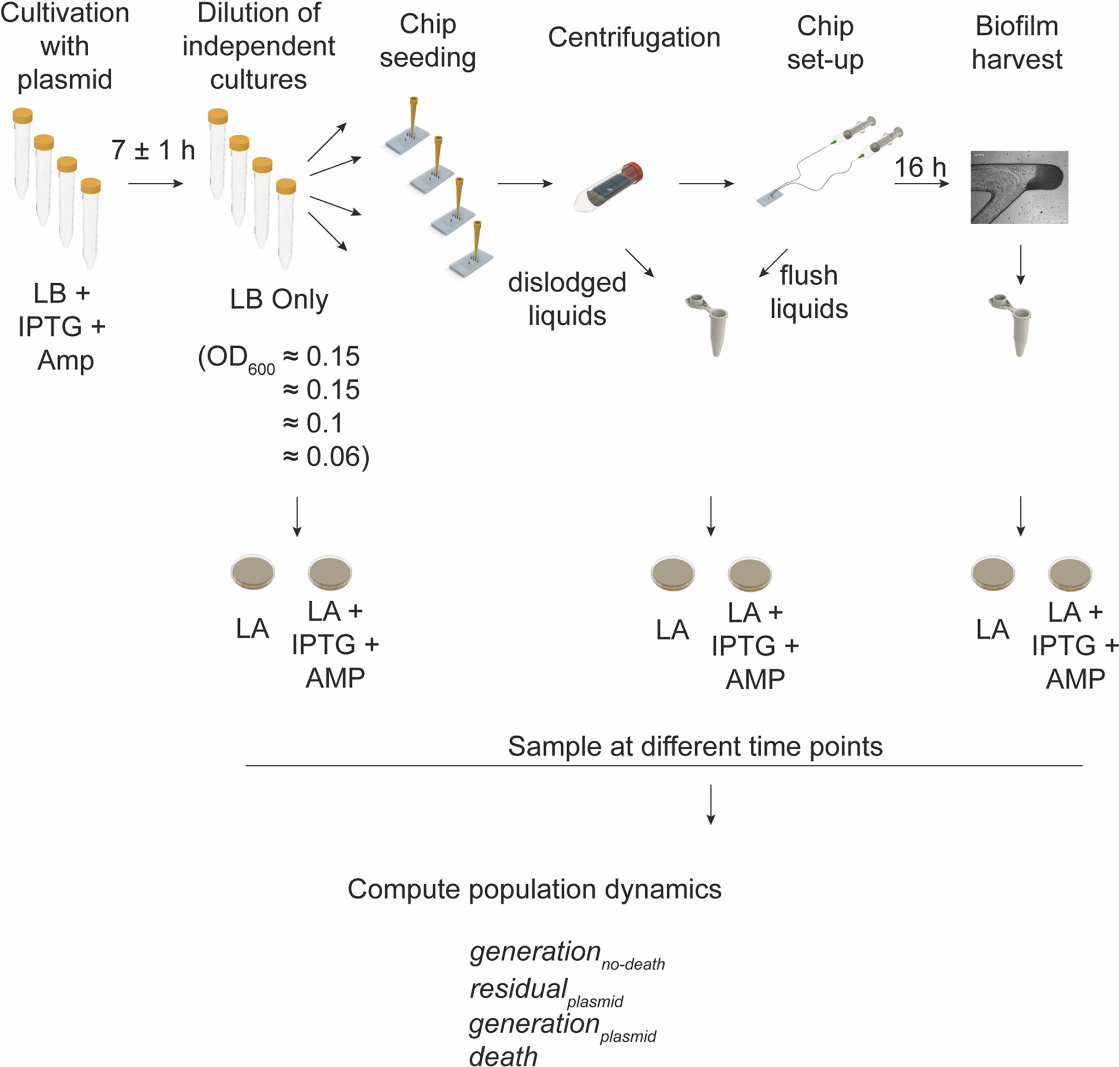
Workflow for determining the number of generations and death events. Schematic illustration of the experimental workflow with independent starting cultures of *E. coli* MG1655 harboring the IPTG inducible pAM34 plasmid. The initial cell growth was with ampicillin selection and added IPTG (enabling replication of the plasmid). Each culture was diluted in fresh growth medium (LB without ampicillin and IPTG) to different optical densities (OD_600_ = 0.06 to 0.15). The diluted cells were used as the inoculum for seeding of four independent chips. Cultivation of biofilms was allowed for 16 h before harvesting of the entire biofilm from the growth chambers. Sampling at different points of the workflow was carried out in order to determine the cell population and plasmid dynamics by plating on selective (LA + IPTG + AMP) and nonselective (LA) growth medium agar dishes. Population dynamics were then estimated from the calculated colony forming units and according to the here described mathematical models (see *Materials and Methods*).

The microfluidic Brimor chip (**Figure 1**) was designed to allow for controlled seeding and long-term growth of bacterial biofilms. Importantly, the system was designed to allow for high-resolution live imaging of fluorescently labelled bacteria in biofilms using confocal microscopy. Each biofilm chip contained two separate biofilm growth chambers (**Figure 1A-1**) connected to outlet and inlet ports (**Figure 1A-2**) *via* outlet and inlet flow channels (**Figure 1A-1**). All growth chambers had an area of 0.687 mm^2^ that held a volume of 25 nL. Both growth chambers in a chip were operated in parallel in all experiments described in this work to allow for simultaneous data collection from one treatment condition and a corresponding control condition (**Figure 1A-2**). A vacuum grid (with a channel height of 100 µm) surrounding the flow channels and growth chambers was added to ensure firm adhesion between the PDMS and the glass slide, to prevent fluid leakage when performing longer experiments, and to prevent bubble formation in the fluidic system (Zhu et al., 2008; Barkefors et al., 2009; Hernández Vera et al., 2019). The entire design was implemented on a 3D-printed mold used for PDMS replica casting (**Figure 1A-3)** and **Figure 1B-3**). Once fabricated, the chip is placed on any microscope of choice with the remaining components of the system connected to the chip (**Figure 1A-4** and **Figure 1B-4**).

Planktonic cells were introduced into the system by pipetting cell suspensions directly into the inlet ports, for both growth chambers, to completely fill the flow channels and the growth chambers. Microfluidic chips loaded with bacteria were placed in a custom-made chip holder to enable centrifugation in a 50 mL tube, to gently pellet bacteria into the biofilm growth chamber. After centrifugation and pelleting of bacteria, the inlet ports were connected to syringes containing growth medium supplemented with or without antibiotics or substrate dye, and the outlet ports were connected to tubing leading to waste collection reservoirs (**Figure 1A-4** and **Supplementary Figure S2**). Harvesting of seeded cells from the growth chambers repeatedly resulted in cfu around 10^4^ to 10^5^. Cultivation of these cells for 16 h as biofilms repeatedly resulted in cfu around 10^6^ to 10^8^. The number of channels and corresponding growth chambers per chip could be increased according to the needs of the user, and the size of the channels connected to growth chamber could also be scaled-up or down utilizing 3D-printed molds or photolithography approaches (Baltekin et al., 2017), respectively.

A number of different biofilm chip designs were experimentally tested and the design that had a 45° angle between the inlet and outlet channel was found to be optimal for biofilm seeding and growth (**Supplementary Figure S3**). We observed efficient partition and harvesting capability of distinct biofilm layers in a controlled fashion (described in detail in next section). Finite element method (FEM) simulations were performed to evaluate and illustrate the fluid flows through the inlet channel, growth chamber, and outlet channel of this optimal chip design (**Figure 2A**) and validated our experimental observations. This led to the addition of a high velocity channel flush in the operational protocol (where the flow rate was set at 100 µL/min for one min) ensuring that any remaining cells outside the growth chamber after seeding were removed from the channels (**Figure 2B**).

### 3.2 Biofilm Formation

Biofilms were grown using strain *E. coli* MG1655 genetically engineered to constitutively express either OFP or CFP to allow for time-lapse imaging of biofilm formation, either in single strain biofilms or in mixed strain biofilms (**Figure 2B** and **Supplementary Movie S2**). The intensities of the OFP and CFP signals were slightly different, and the strongest signals often came from bacteria in the outer edges of biofilms formed in the growth chamber (**Figure 2B**). Most biofilms in the current study were grown for 16 h, but the biofilm chip was also shown to support biofilm growth for 144 h (6 days; **Figure 3A**). Biofilms grown for several days eventually filled up the growth chambers and expanded into the flow channels. The relatively high flow velocity in the flow channels (**Figure 2A**) prevented biofilm overgrowth (specifically beyond the growth chamber area) as the outer loose biofilm layers was continuously removed by the laminar flow, thereby keeping the biofilm in the growth chamber at a relatively stable volume over time (**Figure 3A**).

Staining of biofilms formed from wild-type *E. coli* (DA5438) with CFW that binds to β-1,3 and β-1,4 polysaccharides provided evidence for cellulose production. When compared to a cellulose deficient strain lacking the entire cellulose synthesis pathway (DA64255), the biofilm showed an overall but not easily visible lower fluorescent intensity over the entire growth chamber area within 45 min of the destaining procedure compared to a cellulose producing wild-type strain. This slight difference in CFW intensity was still observed after two hours of destaining. In line with a previous study (McCrate et al., 2013), we also did not observe a difference in CFW intensity when comparing biofilms produced by wild-type *E. coli* to a curli deficient strain DA46932) (**Supplementary Figure S4**). The small difference in cellulose production suggested that the wild-type *E. coli* does shift towards a biofilm lifestyle over time in the chip growth chamber. To confirm this finding, we investigated the integrity of the wild-type strain compared to a quadruple-deficient strain (DA72167) lacking the entire genetic pathways for cellulose, curli, colonic acid and fimbriae synthesis. Indeed, when partitioning the biomass after 16 h of cultivation, we observed cells leaving the growth chamber from the wild-type strain-initiated biofilm containing ECM as chunks. In comparison, the quadrable deficient strain-initiated biofilm dissociated more smoothly from the growth chamber as single cells in response to increased fluid flow (**Figure 3B** and **Supplementary Movie S3**). Taken together, our results show that the wild-type *E. coli* MG1655, in spite of its relatively poor biofilm forming ability, does shift towards a biofilm lifestyle within 16 h of cultivation in the growth chamber of the Brimor chip.

### 3.3 Biofilm Partitioning and Harvesting

Sequential and controlled harvesting of layers of the biofilm from the surface towards the biofilm core region demonstrated by increasing the flow rates in the biofilm flow channels (**Figure 3C** and **Supplementary Movies S4** **,** **S5**). Biofilms grown for longer periods of time (144 h) were more resilient to partitioning and required higher flow rates in order to be efficiently partitioned compared to shorter periods of time (16 h cultivated biofilm).

### 3.4 Number of Generations and Death Events Estimation

We applied the biofilm chip to estimate the number of generations that *E. coli* MG1655 would undergo during 16 h of biofilm formation utilizing a strain harboring the pAM34 plasmid (DA61692, **Figure 4**). We cultivated biofilms in absence of antibiotic and under conditions when the plasmid could not replicate (absence of the inducer IPTG) and estimated the viable cells (as cfu) within the growth chamber during growth of bacteria with and without the plasmid. Between the four independent experimental set-ups with different seeding inoculums, the seeded cells harboring the plasmid was in the order of 8x10^5^ cfu and the final viable number of cells recovered from the 16 h biofilm was approximately 1.1x10^8^ cfu. Therefore, the number of generations computed (not considering death, Eq. 1) had a median of seven generations. From this we then computed the rate of residual replication (*r*) of the plasmid relative to the division rate. This resulted in a median *r* value of 0.8. From this, we were then able to compute the true number of generations of growth to be a median of seven (based on plasmid segregation, Eq. 2) with an experimentally determined death rate of zero (**Supplementary Figure S5**).

### 3.5 Competition Experiments Using Mixed Strain Biofilms

**Figure 5:**
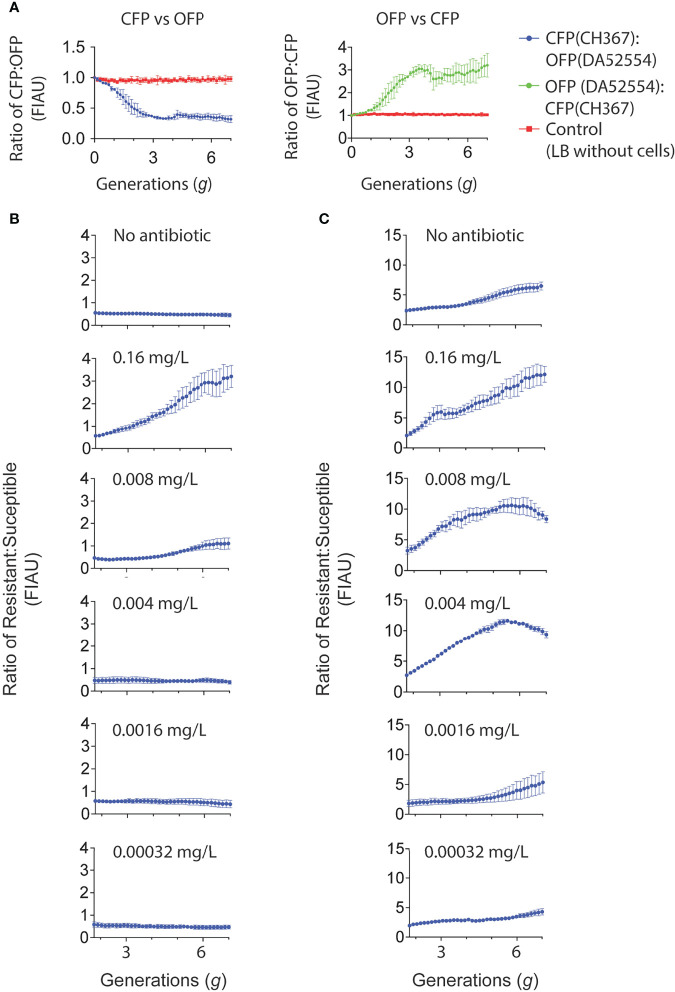
Cultivation of mixed strain biofilms exposed to low concentrations of ciprofloxacin. **(A)** Direct competitions between *E. coli* MG1655 labeled with CFP (CH367) and OFP (DA52554) in mixed strain biofilms. The strains were mixed 1:1 and biofilm growth studied using time-lapse confocal microscopy over a 16 h period. The mean ratio of each of the fluorescence observed from three independent competition experiments as normalized fluorescent intensity arbitrary units (FIAU) are presented for both graphs in **(A–C)** Direct competition of mixed strain biofilms of wild type *E. coli* MG1655 strains (CH367, CFP; DA52554, OFP; as presented in A) and *E. coli* MG1655 with the *gyrA*(S83L) ciprofloxacin resistance mutation to account for strain and fluorescence marker differences (CH368, CFP + *gyrA*(S83L); DA66208, OFP + *gyrA*(S83L)). The ciprofloxacin susceptible and resistant strains were mixed at 1:1 ratio. **(B)** The mean of 15 independent competitions for the resistant strain (CH368) vs. susceptible strain (DA52554) without ciprofloxacin supplemented in growth medium (LB only) is shown, whilst for all remaining graphs the mean of three independent competitions is presented. **(C)** The mean of 18 independent competitions for the resistant strain (DA66208) vs. susceptible strain (CH367) without ciprofloxacin supplemented in growth medium (LB only) is shown. For the ciprofloxacin concentrations 0.016, 0.0016, and 0.00032 mg/L the mean of four independent experiments is shown. The remaining graphs shows the mean of three independent competitions. Standard errors of the mean are indicated for all graphs in **(A–C)**.

**Figure 6:**
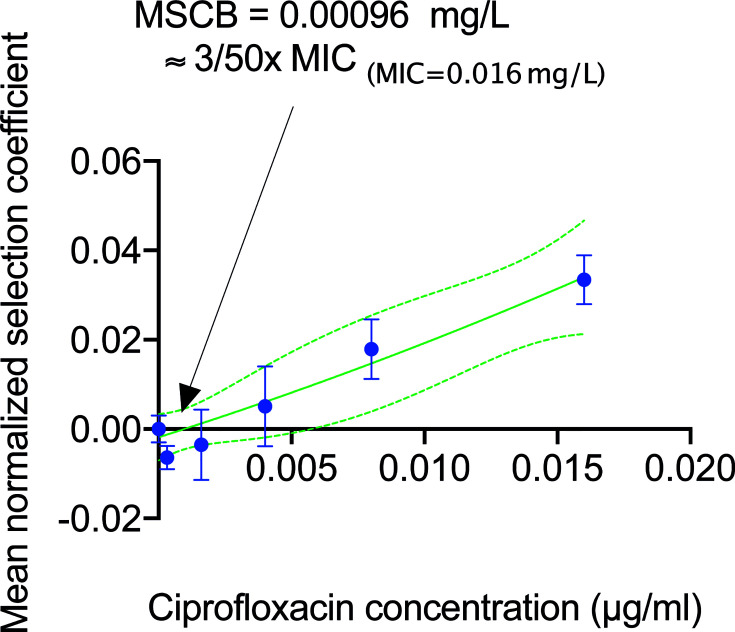
Determination of the minimal selection concentration of ciprofloxacin in biofilms. Mean selection coefficients (blue dots) fitted with a second-degree polynomial fit (indicated as a green line and 95% CI indicated in dashed green lines) as a function of ciprofloxacin concentration determined from direct competitions of mixed strains of ciprofloxacin susceptible and *gyrA*(S83L) ciprofloxacin resistance *E. coli* MG1655 in a 16 h biofilm formation experiments. The minimal biofilm selection concentration (MSCB) was calculated to 0.00096 mg/L. All error bars represent SEM.

We used the biofilm system to determine the effects of sub-MIC levels of ciprofloxacin (a key drug for treatment of urinary tract infections) on selection when cells grew in a biofilm. By establishing a mixed biofilm consisting of fluorescently tagged susceptible and resistant strain (carrying the *gyrA* S83L resistance mutation (Gullberg et al., 2011)), we could in a competition experiment in the biofilm device follow how the ratio of these strains changed in response to antibiotic exposure over a period of 16 h corresponding to seven generations of growth. Control experiments showed that the observed shifts in the overall fluorescence intensity due to exposure to sub-MIC of ciprofloxacin was not dependent on the fluorescence reporters used and had negligible impact on the growth of these strains in forming biofilms. Comparing the computed ratios from the normalized CFP to OFP and the inverse during the four to seven generations (corresponding to 4 to 16 h) there was a slight decrease in the OFP strain (**Figure 5A**). This decrease in the OFP population was likely due to the combined effects from the constitutive promoter driving the expression of the two different fluorescent proteins and the differences between the ECFP and dTomato protein themselves (Eijlander and Kuipers, 2013). Indeed, we observed the same reduced amount of the OFP strain when we grew harvested cells on agar plates (**Supplementary Table S2**). The data presented in **Figures 5B, C** shows changes in the ratio of resistant to susceptible strains as a function of the number of generations of growth at the different concentrations of ciprofloxacin. A negative slope obtained in the absence of ciprofloxacin is a measure of the fitness cost of the resistance mutation, and a positive slope indicates enrichment of the resistant mutant in the population. For each independent competition experiment, we fitted a second-degree polynomial trend between two to seven generations of growth (Ram et al., 2019). This was used to calculate the individual selection coefficient slope (**Supplementary Tables S3**, **S4**) after correction from the selection coefficient slope determined with the parental strain set (**Supplementary Figure S6**). The mean selection coefficient obtained from these experiments are presented as a function of ciprofloxacin concentration, and the intercept where *s* = 0 represents the MSCB, i.e., the concentration of drug where the fitness cost of the resistance is balanced by the antibiotic-conferred selection for the resistant mutant (**Figure 6** and **Supplementary Table S5**). The MSCB for ciprofloxacin was approximately 17-fold lower than the planktonic lifestyle MIC of the susceptible strain, corresponding to an absolute ciprofloxacin concentration of 0.00096 mg/L.

## 4 Discussion and Conclusions

The current study describes the design, fabrication, and utility of a new microfluidic chip for detailed *in vitro* studies of biofilms using time-lapse imaging. We demonstrated the transition of planktonic cells to biofilms, the possibility of controlled partitioning of biofilms, and how the microfluidic chip allows determination of the minimal selective concentration as well as bacterial growth and death rates in biofilms.

A novel potential of our system is the possibility to harvest cultivated biofilms for downstream analysis and re-cultivation in a new chip or other biofilm models. Our results suggest that depending on the particular strain, species and maturity of the biofilm studied, individual harvesting protocol would be required. By optimizing the change in flow rate and time exposed at the high flow rate in the microfluidic chip one could achieve even finer partitioning of biofilms in the growth chamber. The simplicity of microfluidic chip allows investigators the possibility, with high resolution, to determine the heterogeneous differences within a single biofilm without the loss of the spatial resolution. In addition, the cultivation time of biofilms in microfluidic models is often a major issue since contamination and over-growth occur (Subramanian et al., 2020). To the best of our knowledge, the system described in this work is the only microfluidic model that allows a cultivation time as long as 144 hours (6 days) without the introduction of air bubbles to the system thereby disrupting the flow characteristics and biofilm formation and cultivation. As different organisms produce different amounts of EPS and because the amount of EPS increases with age of the biofilm, biofilm age is a major factor in influencing the outcome of an antibiotic therapeutic regimen (Donlan, 2001; Donlan, 2002; Singla et al., 2013; Hall and Mah, 2017).

Mechanical inputs, such as stress, elasticity or compression play a role in the transition from planktonic to biofilm lifestyles, and fluid shear force has been described to drive marine biofilm formation, affect biofilm spatial structure, and assist in regulating virulence during host attachment (Stoodley et al., 2002; Alsharif et al., 2015; Rodesney et al., 2017; Thomen et al., 2017; Catão et al., 2019; Yang et al., 2019). As our chip is microfluidic-based, sheer force actively contributes to the environmental cue and initiates the switch from planktonic to biofilm lifestyle. Compared to straight-flow channel fluidic chips (the majority of dental biofilm systems) the angled channel design connected to a growth chamber of our biofilm chip is likely to enhance this switch in the active growing-front of the biofilm. The remaining part of the biofilm at the depth of the growth chamber had already experienced this force and does not experience constant sheer force. Indeed this phenomenon has been described previously (Rusconi et al., 2010; Rusconi et al., 2011). Owing to the generation of an ECM that forms the physical foundation of the biofilm structure (Flemming and Wingender, 2010), a localized micro-gradient of growth medium could be generated even with continuous fresh medium supplied (Flemming et al., 2016) within this system. Given the porosity of PDMS in the system set-up, as well as the differences in the ECM produced between different bacterial species and even within different strains (López et al., 2010), extensive characterization would be required to capture the macro- and micro-gradient differences within the growth chamber, which is beyond the scope of this work.

Bacteria can become resistant to antibacterial compounds by lateral acquisition of resistance genes or by mutations (Blair et al., 2014). How mutational resistance emerges, spreads and is maintained within a population of bacteria is determined by the interplay of several basic factors, including the biological fitness cost of the resistance gene and the strength of the selective pressure (Hughes and Andersson, 2017). The number of generations and death rate estimations are essential to determine when (i) quantifying the minimal selective concentration, (ii) measuring mutation rates (Frenoy and Bonhoeffer, 2018) and (iii) predicting the evolutionary trajectories of antibiotic resistance (Gullberg et al., 2011). Our experiments demonstrate the possibility to investigate these important parameters in the biofilm chip. Previous work show that the first mutational event during the evolution of ciprofloxacin resistance in *E. coli* is a mutation in the *gyrA* gene (Huseby et al., 2017) and that the MSC for planktonically growing bacteria with the same *gyrA*(S83L) resistance mutation used here is 0.0001 mg/L (Gullberg et al., 2011). This is about 160-fold lower than the MSCB determined in this study The difference in the MSC (planktonic lifestyle) and MSCB (biofilm lifestyle) is not surprising considering that these two growth modes are very different with regard to gene expression, metabolism and physiology (Azeredo et al., 2017). An important future question is to examine the potential generality of this observation, and whether minimal selective concentrations are generally higher in biofilms irrespective of antibiotic class and resistance mechanism.

In principle, the microfluidic biofilm chip presented here is not restricted to only antibiotic compounds but can be used to investigate the effects of any bioactive compounds. Notwithstanding the limitations which accompany the use of a microfluidic approach for biofilm studies (Yawata et al., 2016), bacteria are not the only lifeforms which transition into the biofilm lifestyle and any cell type could be placed in the micro-channels (Flemming and Wuertz, 2019) and we anticipate applications in many areas of microbiology where biofilms are common (Costerton, 1995).

## Data Availability Statement

The original contributions presented in the study are included in the article/**Supplementary Material**. Further inquiries can be directed to the corresponding authors.

## Author Contributions

P-CT: device development, method validation, experiments, data analysis, writing—original draft, review, and editing. OE: device design and development, and writing—review and editing. JS: experiments and data analysis—review and editing. JK and DA: device design, supervision, funding resources, and writing—review and editing. All authors have read and approved the final version of the manuscript.

## Funding

This work was supported by grants to JK from the Uppsala Antibiotic Centre, and to DA from the Swedish Research Council (Grant 2017-01527) and the Thon Foundation.

## Conflict of Interest

A patent application comprising the chip design and the here described method has been filed by P-CT, OE, and JK under number PCT/SE2019/050635. JS is currently employed by Corline Biomedical AB. The company had no part in the design, performance, or analysis of data in the study.

The remaining author declares that the research was conducted in the absence of any commercial or financial relationships that could be construed as a potential conflict of interest.

## Publisher’s Note

All claims expressed in this article are solely those of the authors and do not necessarily represent those of their affiliated organizations, or those of the publisher, the editors and the reviewers. Any product that may be evaluated in this article, or claim that may be made by its manufacturer, is not guaranteed or endorsed by the publisher.
