# Supplementary material for: A Microfluidic Chip for Studies of the Dynamics of Antibiotic Resistance Selection in Bacterial Biofilms
Source: Front Cell Infect Microbiol. 2022 May 10;12:896149. doi: 10.3389/fcimb.2022.896149 (PMC9128571; doi:10.3389/fcimb.2022.896149)
Supplement: Supplementary file 1 [file DataSheet_1.docx]

Supplementary Material

Supplementary Table S1. List of bacterial strains and genotypes used in this study.

| **Strain** | **Genotype** | **Reference/Source** | **MIC (mg/L)** | **Comment** |
| --- | --- | --- | --- | --- |
| DA5438 | Wild type MG1655 (parent) | DA Strain Collection | 0.016 | Biofilm competition |
| DA64255 | DA5438, ∆*yhjR* ∆*bcsQABZCEFG* | This study | not determined | Cellulose operon knockout |
| DA46932 | DA5438, ∆*csgCABDEFG* | This study | not determined | Curli operon knockout |
| DA43034 | DA5438, ∆*fimBEAICDFGH* | This study | not determined | Fimbriae operon knockout |
| DA72161 | DA5438, ∆*cpsBG* ∆*fcl* ∆*gmd* ∆*gmm* ∆*wcaABCDEFIJKLM* ∆*wza* ∆*wzb* ∆*wzc* ∆*wzxC* | This study | not determined | Colonic acid biosynthesis knockout |
| DA72167 | DA5438, ∆*yhjR* ∆*bcsQABZCEFG* ∆*csgCABDEFG* ∆*cpsBG* ∆*fcl* ∆*gmd* ∆*gmm* ∆*wcaABCDEFIJKLM* ∆*wza* ∆*wzb* ∆*wzc* ∆*wzxC* ∆*fimBEAICDFGH* | This study | not determined | Cellulose, curli, colonic acid biosynthesis, fimbriae knockout |
| DA61692 | DA5438, pAM34 | DA Strain Collection | not determined | Generations & death |
| LM179 | Wild type MG1655 (parent) | 1 | 0.016 | Biofilm competition |
| CH367 | LM179, ∆*lac*::*FRT galK*::*ecfp*-*bla* | 1 | 0.016 | Biofilm competition |
| CH368 | LM179, ∆*lac*::*FRT galK*::e*cfp*-*bla yfaH*::*FRT gyrA*(S83L) | 1 | 0.5 | Biofilm competition |
| DA52554 | DA5438, ∆*IS150*::*dTomato* pSIM5-tet | This study | 0.016 | Biofilm competition |
| DA66208 | DA5438, ∆*IS150*::*dTomato* *gyrA*(S83L) | This study | 0.5 | Biofilm competition |

**Supplementary Figure S1.** Fabrication of the microfluidic chip Brimor. Steps taken in order to fabricate the microfluidic chip with polydimethylsiloxane (PDMS) is numbered from 1 to 7. Illustrations and photographs of the various steps are shown.

**Supplementary Figure S2.** Operation workflow of the microfluidic system. Schematic illustration of the experimental workflow starting with standard planktonic cultivation of bacterial cultures in test tubes before dilution in fresh growth medium. The optical density of the diluted culture was determined. The growth chamber was then filled with diluted cell suspension via the inlet port until the entire inlet port, inlet channel, growth chamber, outlet channel, outlet port was filled with liquid. The chip was then placed in a custom-made 3D-printed chip holder to allow for centrifugation in a standard 50 mL centrifugation tube to gently pellet the bacteria towards the bottom of the growth chamber. Thereafter, the chip is connected to a syringe pump and placed securely on a microscope slide holder already fastened on the microscope stage before the vacuum is connected to the vacuum port on the biofilm chip. Finally, the syringe pump is started at specific flow rate and time-laps imaging performed.

**Supplementary Figure S3.** Evaluation of alternative channel designs. For all chip architectures (A-G), *E. coli* MG1655 were seeded into the growth chambers (area indicated with *) and cultivated as biofilms in LB growth medium for 18.5 h at room temperature with a flow rate set to 5 µL/min. An initial inflow rate of 1000 µL/min for 20 s and then >1999 µL/min was applied in all designs when evaluating the stratification and harvesting capability. (A-C) All channels had a width of 400 µm and z-height of 100 µm. (D-G) Channels on the right side of the growth chamber had a width of 400 µm, whilst the channels on the left side of the cultivation chamber had a width of 200 µm; both had a z-height of 100 µm. All scale bars on images represent 500 µm.

**Supplementary Figure S4.** Calcofluor white (CFW) staining of cellulose (a) and curli (b) knockout *E. coli* biofilms. Biofilms grown for 16 h were stained with CFW to detect extra-cellulose production from three different founding strains: wild-type (wt) *E. coli* MG1655, an isogenic cellulose deficient strain (the *bcs* operon encoding the entire cellulose synthesis pathway was genetically removed from the chromosome) and an isogenic curli deficient strain (the *csg* operon encoding the entire curli component was genetically removed from the chromosome). For all panels in (a) & (b) the first row is false-colored red fluorescence images from CFW staining and the second row is the corresponding brightfield images. All scale bars represent 500 µm.

**Supplementary Figure S5.** Estimations of the number of generations and death events of biofilms cultivated in Brimor.

**Supplementary Table S2.** Entire biofilms from 4 independent chips were harvested and collected at 0, 4, 8, and 16 h. Samples were plated on LB agar and LB supplemented with 0.15 mg/L ciprofloxacin agar to determine the resistant and susceptible ratios via traditional agar plating and counting the number of colonies observed to determine the cfu.

**Supplementary Table S3.** Individual selection coefficient determined for the independent competition experiments when CH368 and DA52554 were cultivated together in Brimor chip as biofilms in LB broth supplemented with varying concentrations of ciprofloxacin. Each colored s-values are related to the independent experiment performed.

**Supplementary Table S4.** Individual selection coefficient determined for the independent competition experiments when DA66208 and CH367 were cultivated together in Brimor chip as biofilms in LB broth supplemented with varying concentrations of ciprofloxacin. Each colored s-values are related to the independent experiment performed.

**Supplementary Figure S6.** Individual selection coefficient determined for the independent competition experiments when DA52554 and CH367 were cultivated together in Brimor chip as biofilms in LB broth as the cost of the different fluorescent markers used in the strains.

**Supplementary Table S5.** Average s-values determined from s-values obtained in supplementary Table S2 and Table S3. Blue colored values denote s-values obtained from the strain pair CH368 and DA52554, whilst red colored values denotes s-values obtained from the strain pair DA66208 and CH367.

**Supplementary Movie S1.** Oxygen corona plasma treatment to activate the PDMS and glass surfaces. Treatment is done is done with exposure to plasma for 1 min across the entire surface of the PDMS and the glass slide.

**Supplementary Movie S2.** Formation of *E. coli* biofilms in the growth chamber. *E. coli* MG1655 chromosomally tagged with cyan florescent protein (CH367) or orange fluorescent protein (DA52554) was cultivated in the top and bottom growth chambers, respectively for 16 h to demonstrate biofilm growth in the Brimor chip.

**Supplementary Movie S3.** Integrity of quadruple deficient and wild-type *E. coli* biofilms grown for 16 h. The wild-type *E. coli* MG1655 was cultivated in the bottom growth chamber. The isogenic quadruple deficient strain (DA72167) lacking the entire genetic pathways for cellulose, curli, colonic acid and fimbriae synthesis was grown in the top chamber. Partitioning was executed by increasing the flow rate from 5 µL/min to 1000 µL/min for 10 sec.

**Supplementary Movie S4.** Partitioning and harvesting of an *E. coli* biofilm grown for 16 h. The first biofilm layer was partitioned and harvested by increasing the flow rate from 5 µL/min to 1000 µL/min for 10 sec, and the liberated biofilm layer was then flushed out using a flow rate of 5 µL/min for 5 minutes. The second layer was partitioned using a flow rate of 1000 µL/min for 20 sec. The remaining biofilm was harvested with a flow rate >1999 µL/min for 10 sec. Scale bars represent 500 µm.

**Supplementary Movie S5.** Partitioning and harvesting of an *E. coli* biofilm grown for 144 h. The first biofilm layer was partitioned and harvested by increasing the flow rate from 5 µL/min to 1000 µL/min for 47 sec, and the liberated biofilm layer was then flushed out using a flow rate of 5 µL/min for just 4 min. The second layer was partitioned using a flow rate of >1999 µL/min followed by and the remaining layer of the biofilm was harvested with a flow rate >1999 µL/min. Scale bars represent 500 µm.

Reference

1	E. Gullberg, S. Cao, O. G. Berg, C. Ilbäck, L. Sandegren, D. Hughes and D. I. Andersson, PLoS Pathog., 2011, 7, e1002158.
